# Fungal keratitis complicating the diagnosis of *Acanthamoeba* keratitis

**DOI:** 10.1016/j.mmcr.2024.100687

**Published:** 2024-12-07

**Authors:** Mehrnaz Atighehchian, Alireza Latifi, Zohreh Nozarian, Fahimeh Asadi Amoli, Mehran Zarei-Ghanavati

**Affiliations:** a Farabi Eye Hospital, Tehran University of Medical Sciences, Tehran, Iran; b Department of Medical Parasitology and Mycology, School of Public Health, Tehran University of Medical Sciences, Tehran, Iran; c Pathology Department, Farabi Eye Hospital, Tehran University of Medical Sciences, Tehran, Iran

**Keywords:** Fungal keratitis, Acanthamoeba, Infiltration

## Abstract

A 42-year-old woman was referred to an emergency department. She had an unresponsive corneal ulcer that was initially diagnosed as *Herpes simplex* virus keratitis. Later, the microbiological studies revealed fungal keratitis. Although the patient was given topical antifungal medication, the clinical presentation did not support improvement. Despite using antifungal medication, the infiltration continued to progress, and the patient underwent therapeutic penetrating keratoplasty (T-PKP). Corneal tissue was collected and sent for histopathologic and molecular examination. The results revealed the presence of both *Acanthamoeba* T4 subgroup and *Fusarium* sp. This case emphasizes the importance of considering *Acanthamoeba* infection in progressive and non-responsive infectious keratitis, especially fungal specimens. Polymerase chain reaction (PCR) is an appropriate laboratory molecular diagnostic test for accurate diagnosis *of Acanthamoeba* keratitis.

## Highlights

• Acanthamoeba keratitis (AK) is a serious infection that can lead to significant visual damage and is challenging to diagnose due to early symptoms resembling other forms of keratitis.
• AK coinfections with fungi are more common than recognized and can occur even in non-contact lens users, often requiring therapeutic keratoplasty.
• Early diagnosis and treatment are crucial, with PCR being essential for accurate detection. This case highlights the need for considering AK in non-responsive keratitis.

## 1 Introduction

A wide range of microorganisms cause infectious keratitis. *Acanthamoeba* keratitis (AK) is a serious eye infection that can lead to significant visual damage [1]. The most common risk factors for AK are wearing contact lenses, including exposure to contaminated water, dust, and ocular trauma [1,2]. Diagnosing can be challenging as its symptoms in the early stage are similar to other forms of infectious keratitis, such as *Herpes Simplex* Virus (HSV) keratitis. Furthermore, the treatment is complex, significantly when the deeper layers of the cornea are affected. Early diagnosis and appropriate treatment are crucial for an excellent visual outcome [[2], [3], [4]].

It is important to note that *Acanthamoeba* coinfection can develop in eyes with another advanced infectious keratitis [5,6]. Although coinfections of *Acanthamoeba* and fungi have been reported in patients, especially in contact lens users [4,[7], [8], [9]], these mixed-infectious are much more frequent and are not restricted to contact lens users [10]. Such cases can cause advanced keratolysis and require therapeutic keratoplasty to control infiltration [[9], [11]]. This case report describes a patient with an unusual history and clinical presentation of coinfection *Acanthamoeba* and fungal keratitis, which has been proven by molecular and histopathology examinations and emphasizes the importance of using real-time PCR for detecting *Acanthamoeba*.

## 2 Case description

A 42-year-old farmer woman with an unresponsive corneal ulcer was referred to our emergency department. She experienced redness, pain, photophobia, and decreased vision for four weeks. She had been treated for HSV keratitis with oral acyclovir at other centers, but her symptoms worsened from the initial presentation. She had no prior history of contact lens use or eye trauma, but she reported an unclear history of exposure to dust while farming.

During our examination (day 0), we noticed that the patient's vision was impaired and detected hand motion. Slit-lamp examination revealed mild eyelid swelling, mucopurulent discharge, and diffuse bulbar conjunctival injection throughout the left eye. The cornea showed a grayish-white infiltration with an irregular margin at the center, which measured 6 × 7 mm and extended up to the mid-stromal level. We also noted small diffuse keratic precipitates, multiple satellite lesions at the periphery of infiltration, and mild stromal edema adjacent to the infiltrate. Corneal sensations did not reduce. We observed a 10 × 10 mm corneal epithelial defect with a positive fluorescein stain. B-scan ultrasonography did not show any involvement in the vitreous or retina.

**Fig. 1:**
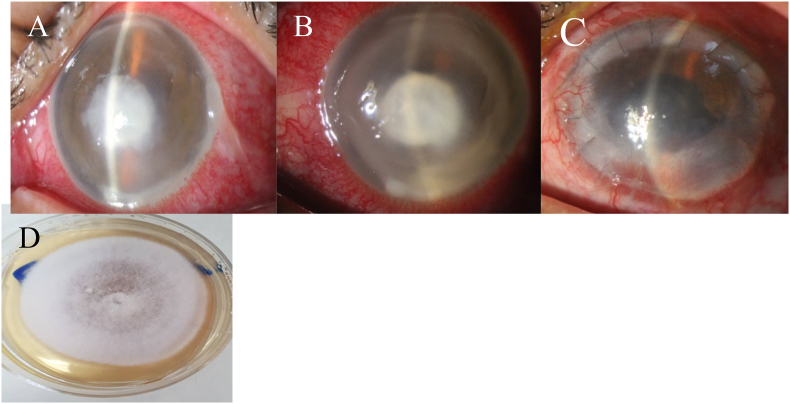
(A) Slit lamp examination, shows 7 × 7 mm gray-white feathery margin stromal infiltrate at center of cornea. (B) On the 12th day of the treatment, deep stromal infiltration, diffuse corneal edema, and hypopyon were progressed. (C) Four months after T-PKP. (D) *Fusarium* spp. grew on Sabouraud dextrose agar.

Upon admission to the hospital, corneal scrapings were taken from the base and edge of the ulcer. The samples were sent for microbiological investigations, including potassium hydroxide (KOH) wet mount preparation, gram smear, and cultures on blood agar, chocolate agar, and Sabouraud dextrose agar. The culture showed *Fusarium* sp. *(day 5),* but bacterial cultures and a PCR test for HSV were negative (Fig. 1). In vivo confocal microscopy (IVCM) was unavailable, even though the diagnosis was confirmed with laboratory tests and deemed necessary.

Due to the concerning history of exposure to planet dust, it is crucial to proceed with caution. Based on the microbiological results, the patient has been diagnosed with definite fungal keratitis. The prescribed treatment included topical voriconazole 1 % to be applied every hour, levofloxacin four times a day, and oral voriconazole 200 mg to be taken twice daily. Unfortunately, natamycin eye drops were not available.

After twelve days, the clinical presentation did not support a response. The corneal edema and infiltration had grown to a size of 7 × 7 mm. The patient also developed an endothelial plaque, fungal ball, fibrin reaction, and a 3mm layer of hypopyon. Stromal corneal edema and necrosis increased and affected the rest of the cornea (Fig. 1).

Hence, the significant stromal keratolysis and peripheral limbal involvement necessitated an early T-PKP with cryotherapy and intra-cameral voriconazole injection. It was not advisable to delay intervention to see if other antifungal medications would be effective.

**Fig. 2:**
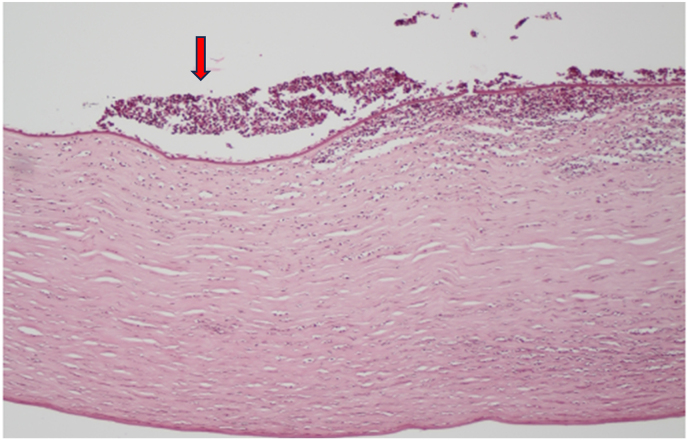
Microscopic examination of H/E-stained slide shows corneal ulceration and neutrophilic infiltration (Arrow) magnification X100. Microscopic features of Hematoxylin and eosin (H/E) stained slides on corneal biopsy include ulceration, neutrophilic infiltrates and presence of characteristic double-walled cystic structure in the corneal stroma with various states of degeneration and also the presence of spectated fungal hyphae, both organisms were positive in Periodic Acid-Schiff (PAS) special staining.

**Fig. 3:**
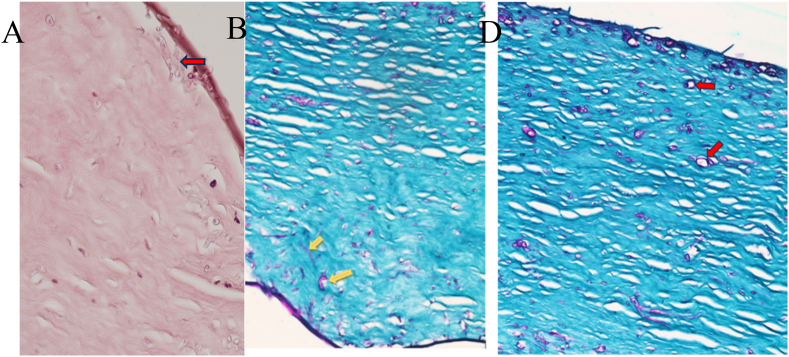
A and B, Microscopic examination of H/E and PAS-stained slides show spectated fungal hyphae (Arrow) magnification X400. C, *Acanthamoeba* cysts.

During surgery (day 13), the corneal sample was taken to analyze the presence of *Acanthamoeba*, bacteria, and fungi using histopathology, microbiological, and PCR testing. The histopathology results showed positive for *Acanthamoeba* cysts and fungal elements. (Fig. 2, Fig. 3), and PCR confirmed *Acanthamoeba*. So, after T-PKP, topical and systemic voriconazole were continued, and polyhexamethylene biguanide (PHMB) 0.02 % was administered hourly. After 6 days successful treatment, the patient was discharged without any further complications. The best-corrected visual acuity (BCVA) was 1.0 LogMAR at six months of follow-up and there was no recurrent infiltration in the graft.

### 2.1 Confirmation of acanthamoeba by molecular method and genotyping

The cornea specimen was transferred to phosphate buffer saline (PBS) for culture and PCR examination.

### 2.2 Culture

Briefly, 100 μL of sample sediment were inoculated onto 1.5 % non-nutrient agar (NNA) overlaid with *Escherichia coli* and incubated at room temperature. To detect the outgrowth of the acanthamoeba trophozoites and cysts, the plate was checked every day for approximately two weeks using an inverted microscope 1.

### 2.3 DNA extraction, PCR, and DNA sequencing

The part of the specimen was used for the molecular technique. DNA extraction was performed without modification using the MagNA Pure LC DNA Isolation Kit I (Roche Germany). Two specific primer pairs were used to detect *Acanthamoeba* spp. A pair of primers JDP1 forward (5′-GGCCCAGA TCGTTTACCGTGAA-3′) and JDP2 reverse (5′-TCTCACAAGCTGCTAGGGAGTCA-3′) was applied to amplify a fragment of 450–500 bp of ASA. S1 region in18S rRNA gene. The conventional PCR reaction by both primer pairs was performed on the thermocycler (PEQLAB peq STAR Thermal Cycler) using a ready-made mixture of Amplicon (Taq DNA Polymerase Master Mix RED, Denmark) in a 25-μl final volume containing 5μl of DNA and 0.3μM concentration of each primer by standard procedure. Thermal cycling conditions were as follows: initial denaturation at 94 °C for 5 min, 35 cycles of denaturation at 94 °C for 35 S, annealing at 56 °C for 45 S, and extension at 72 °C for 60 S and a final extension step at 72 °C for 10 min. To visualize PCR products, they were electrophoresed on 1 % (w/v) agarose gel, stained with green viewer substance (1 μl/10ml), and then the presence of bands (nearly 500bp) was confirmed under UV light using a Gel-doc Image Analyzer 12.

### 2.4 Sequencing analysis

To determine the genotype, a 500-bps fragment of ASA. S1 region was Amplified from the 18s rRNA gene of Acanthamoeba strains by JDP primers. Then, the gel was purified (PCR products purification kit, parstous Corporation, Iran, Cat. No. A101221) and finally sequenced by Codon Company (Iran). The DNA sequences were edited manually with Chromas 2.6.6 software and compared with the BLAST Gen Bank database.

## 3 Result of culture and molecular tests

The culture was not positive on the 14th day based on the defined gold standard diagnostic method. JDP-PCR detected the *Acanthamoeba* parasite on 14th day. The sequencing results identified that the strain belonged to the T4 genotype, with a more than 95 % similarity on the 21st day. The sequencing of isolates was deposited in the GEN BANK DATABASE under (Accession number: PP951901) [12].

## 4 Discussion

*Acanthamoeba* is an opportunistic pathogen and is classified into 23 genotypes. The most common types of *Acanthamoeba* associated with keratitis are *A. castellani*, *A. culbertsoni*, and *A. polyphaga*. Additionally, fungal keratitis is a destructive infection and causes a variety of clinical symptoms, ranging from a superficial, feathery infiltration to a deep endothelial plaque pattern [1,13].

The clinical features of AK may include epithelium erosion, ring-shaped stromal infiltration, perineuritis, and scleritis. However, diagnosing this condition can be difficult and requires clinical suspicion, the use of laboratory tests, and imaging techniques [4,8,14].

The confocal biomicroscopy has made it possible to detect *Acanthamoeba* double wall cysts. Also, microbiological strains, culture media, and immunofluorescence are essential in achieving it [13,14]. In recent years, the diagnosis of AK has been improved through the development of nucleic acid amplification tests. Previous studies have found conventional PCR assays more sensitive than microscopic and microbiologic examinations for detecting acanthamoeba in ocular samples [[13], [14], [15]]. Real-time PCR has now emerged as rapid molecular testing for clinical samples, improving the accuracy of acanthamoeba diagnosis [[13], [14], [15], [16]]. In the present report, non-nutrient agar with *E. coli* culture medium was negative, and we used real-time PCR and genome sequencing to confirm *Acanthamoeba*. The T4 genotype is most commonly associated with clinical disease and is isolated from environmental samples [16]. Our study identified the T4 genotype of *Acanthamoeba* using DNA sequencing techniques.

Despite the diagnostic methods, some initially misdiagnosed as bacterial or viral infections may happen in AK similar to this case. In the early stages of infection, AK is characterized by the presence of dendritic ulcers that resemble those found in HSV keratitis. However, employing molecular diagnostic methods can aid in distinguishing this condition from others [17]. In this study, the patient was initially misdiagnosed with HSV keratitis, leading to treatment with antiviral medications. Unfortunately, the patient's condition worsened as a result and follow-up testing using polymerase chain reaction (PCR) analysis provided definitive results that not only excluded the initial diagnosis but also revealed a different underlying issue that had previously been overlooked.

In addition, anticipating coinfections is necessary for early diagnosis and appropriate treatment because AK may progress faster, especially with fungal coinfection [4]. The coexistence of *Acanthamoeba* with fungi is much more prevalent than is currently realized and has been reported in the literature [1,3,4,7,15,17,18]. Fungi may serve as a nutrient for the amoeba, leading to faster keratolysis. Therapeutic keratoplasty may be required in such cases, and the visual outcomes are poor [[19], [20], [18]]. Both fungal and *AK* are associated with contact lens use, and current literature shows an increasing trend in both types of infections in contact lens users [4].

AK is a rare condition seen in individuals who do not use contact lenses, and cases have been linked to environmental exposure or trauma to the cornea [21]. For example, one study documented a 34-year-old woman with no history of contact lens use who developed AK, likely due to exposure to contaminated water. This case illustrates that *Acanthamoeba* can infect the cornea even in the absence of typical risk factors, particularly if there is any compromise to the cornea's integrity, such as micro-injuries or pre-existing eye conditions. The treatment for this condition was complex and involved *anti*-*Acanthamoeba* therapy. Ultimately, surgical intervention was necessary due to the advanced stage of the infection and the organism's resistance to standard treatments [21,22].

Additionally, fungal keratitis is more prevalent in tropical areas, which means *Acanthamoeba*-fungal coinfections could also be more common in those regions [4].

Raghavan et al. reported *Pythium insidiosum* and *Acanthamoeba* keratitis in a contact lens user [6]. Lee et al. reported simultaneous *Acanthamoeba* and *Fusarium* keratitis associated in contact lens solution and silicone hydrogel contact lens user. They concluded that atypical infectious diseases must be considered in infectious keratitis with poor response to treatment in the contact lens wearer [7]. On the other hand, Gupta et al. showed *Acanthamoeba* coinfections are much more frequent and are not restricted to contact lens users. Slade et al. reported *Acanthamoeba* and fungal keratitis in a woman with a history of intrastromal corneal ring segment implants. They concluded that the atypical sources of AK should be considered in patients with chronic corneal ulcers and a history of ocular surgery [23].

First-line therapy for AK includes topical solutions of biguanides and diamidines. Voriconazole, a triazole antifungal medication, has been reported to inhibit ergosterol biosynthesis in *Acanthamoeba*, which reduce trophozoite proliferation, and prevents encystation. In some cases, the topical application of voriconazole has proven effective, even in instances where traditional AK therapies were resistant [24]. Although topical voriconazole 1 % appears to be an effective option for treating AK, the first-line therapies are PHMB 0.02 % and propamidine isethionate 0.1 % [13,17,24]. In the present report, the patient was treated with topical voriconazole 1 %, but the stromal keratolysis progressed. So, early diagnosis and awareness of co-infections and prompt initiation of first-line medications are crucial for controlling infiltration.

Our study showed a patient with an unusual clinical presentation for AK with a *Fusarium* co-infection. The clinical feature in the initial examination was not specific for *Acanthamoeba* and non-nutrient agar with *E. coli* culture medium was negative. In contrast to the classical grayish ring infiltrate in pure *Acanthamoeba* cases, patchy stromal infiltration is presented in this report. In some previous studies, coinfection of *Acanthamoeba* and fungal species was initially diagnosed together by histopathological or molecular examination. Other reports initially diagnosed *Acanthamoeba*, and coinfections with fungi were confirmed [1,3,4,7,17,19]. However, in our patient, the identification of *Fusarium* sp. complicated the diagnosis of AK, and the diagnosis of *Acanthamoeba* T4 genotype was based on PCR and histopathology of removed tissue.

*In conclusion, Acanthamoeba* coinfections are more common than previously acknowledged. This coinfection should be expected in cases where *Acanthamoeba* is suspected or medically unresponsive keratitis. Hence, repeat microbiological assessments, confocal microscopy, and other molecular tests should be performed to establish the diagnosis. This case emphasizes the importance of considering *Acanthamoeba* infection in progressive fungal corneal ulcer cases despite typical antifungal treatments.

## CRediT authorship contribution statement

**Mehrnaz Atighehchian:** Writing – original draft. **Alireza Latifi:** Methodology. **Zohreh Nozarian:** Investigation. **Fahimeh Asadi Amoli:** Methodology. **Mehran Zarei-Ghanavati:** Writing – review & editing, Supervision.

## Declaration of competing interest

The authors declare that they have no known competing financial interests or personal relationships that could have appeared to influence the work reported in this paper.
